# Construction of a potential microRNA and messenger RNA regulatory network of acute lung injury in mice

**DOI:** 10.1038/s41598-022-04800-3

**Published:** 2022-01-17

**Authors:** Yufeng Zhang, Weilong Jiang, Qingqing Xia, Jinfeng Lin, Junxian Xu, Suyan Zhang, Lijun Tian, Xudong Han

**Affiliations:** 1 Department of Respiratory Medicine, Jiangyin Hospital of Traditional Chinese Medicine, Jiangyin Hospital Affiliated to Nanjing University of Chinese Medicine, Jiangyin, 214400 China; 2 Department of Critical Care Medicine, Nantong Third People’s Hospital, Nantong University, Nantong, 226001 China

**Keywords:** Respiratory tract diseases, Gene regulatory networks

## Abstract

Acute lung injury (ALI) is a life-threatening clinical condition associated with critically ill patients, and the construction of potential microRNA (miRNA) and messenger RNA (mRNA) regulatory networks will help to fully elucidate its underlying molecular mechanisms. First, we screened fifteen upregulated differentially expressed miRNAs (DE-miRNAs) and six downregulated DE-miRNAs from the Gene Expression Omnibus (GEO) database. Then, the predicted target genes of the upregulated and downregulated DE-miRNAs were identified from the miRNet database. Subsequently, differentially expressed mRNAs (DE-mRNAs) were identified from the GEO database and subjected to combined analysis with the predicted DE-miRNA target genes. Eleven target genes of the upregulated DE-miRNAs and one target gene of the downregulated DE-miRNAs were screened out. To further validate the prediction results, we randomly selected a dataset for subsequent analysis and found some accurate potential miRNA-mRNA regulatory axes, including mmu-mir-7b-5p-Gria1, mmu-mir-486a-5p-Shc4 and mmu-mir-486b-5p-Shc4 pairs. Finally, mir-7b and its target gene Gria1 and mir-486b and its target gene Shc4 were further validated in a bleomycin-induced ALI mouse model. We established a potential miRNA-mRNA regulatory network of ALI in mice, which may provide a basis for basic and clinical research on ALI and advance the available treatment options.

## Introduction

Acute lung injury (ALI) and its more severe form acute respiratory distress syndrome (ARDS) are life-threatening clinical conditions associated with critically ill patients and have high morbidity and mortality rates worldwide^1^. ALI/ARDS is characterized by lung epithelial and endothelial cell injury, with increased permeability of the alveolar-capillary membrane, leading to pulmonary edema, severe hypoxia, and difficulty with ventilation^2^. The common causes of ALI/ARDS are sepsis, severe trauma, massive blood transfusion, pneumonia, aspiration of gastric contents, and toxicity from certain types of drugs. The complex pathophysiology of ALI/ARDS seems to provide a wide range of targets that offer numerous therapeutic options^1,2^. However, despite extensive studies on ALI/ARDS pathophysiology and treatment, no effective pharmacotherapies are available^3^.

A range of microRNAs (miRNAs), recently determined by high-throughput screening studies in human and animal models, play a pivotal role in the pathophysiology of ALI/ARDS^4,5^. MiRNAs are small noncoding RNAs ranging in size from 18 to 24 nucleotides that can regulate the expression of specific genes by inhibiting the translation of target messenger RNAs (mRNAs) or by targeting complementary mRNAs for degradation^6,7^. In addition, circulatory miRNAs are beneficial biomarkers and some pharmacotherapeutic targets^8^. This is revolutionary for syndromes that have neither measurable disease indicators nor targeted treatment. Currently, no miRNA-based therapies are available for ALI/ARDS, but therapies targeting miRNAs have reached phase II clinical trials for the treatment of some cancers ^5,9,10^. Further studies may reveal some unique miRNA profile patterns that can serve as biomarkers or targets for ALI/ARDS.

**Figure 1:**
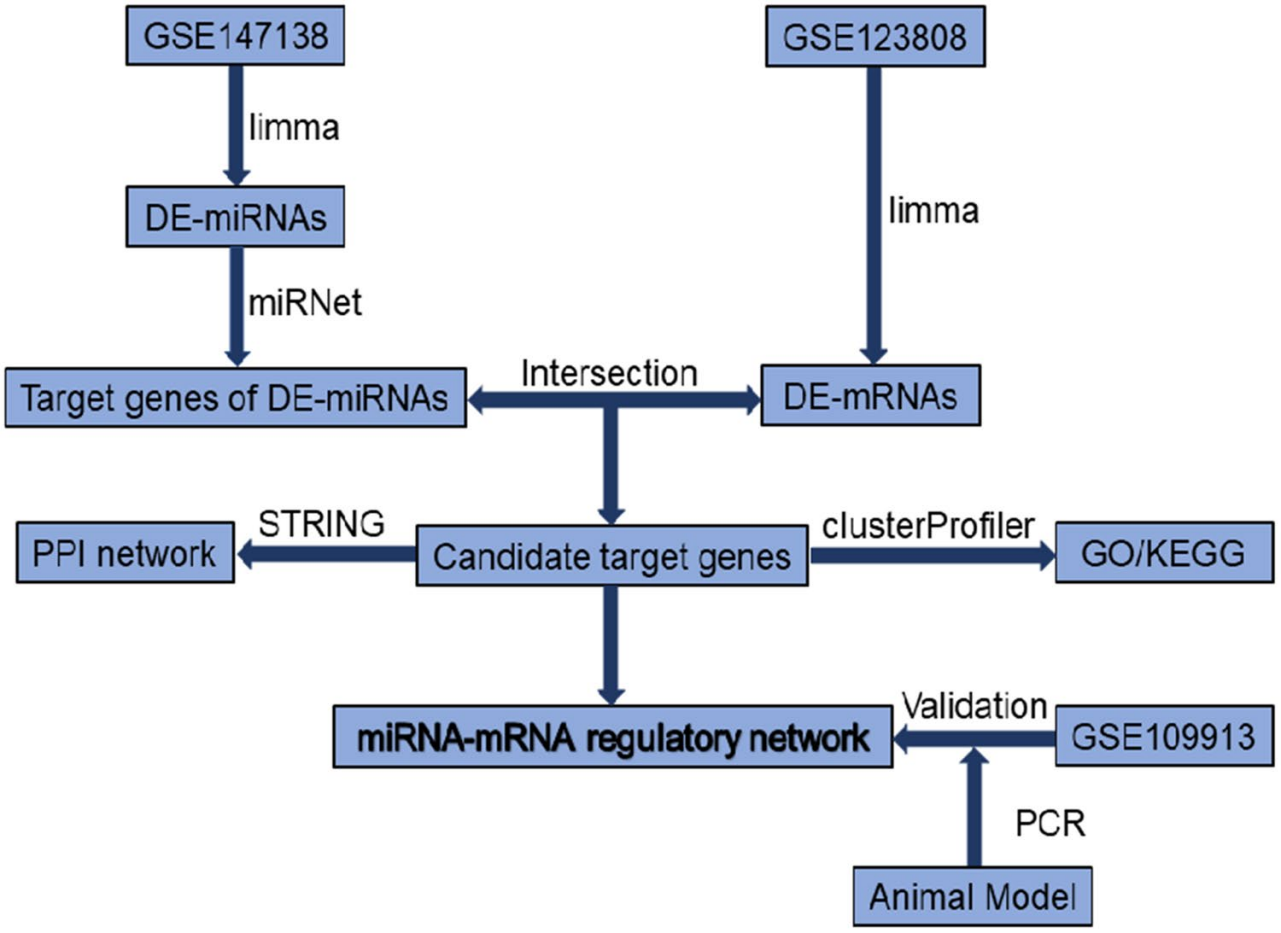
The flowchart of our study.

Because of the complex and heterogeneous mechanisms of human ARDS, a rat model of ARDS induced by saline lavage and mechanical ventilation was used to construct a miRNA and mRNA microarray and thereby identify miRNA-mRNA interactions^11^. The bleomycin-induced ALI mouse model is widely applied because it is characterized by an inflammatory response and alveolar epithelia leading to excessive matrix deposition^12–14^. However, no miRNA and mRNA regulatory network of bleomycin-induced ALI in mice has been constructed. In this study, we searched datasets of bleomycin-induced ALI in mice by accessing the network database. We first screened differentially expressed miRNAs (DE-miRNAs) in bleomycin-treated lung tissues compared with normal lung tissues in mice. Then, we predicted the potential target genes of the DE-miRNAs using network database resources. Next, differentially expressed mRNAs (DE-mRNAs) between bleomycin-treated lung tissues and normal lung tissues were obtained by analyzing the mRNA dataset. Subsequently, candidate target genes were identified, a protein–protein interaction (PPI) network was constructed, and Gene Ontology (GO) functional enrichment and Kyoto Encyclopedia of Genes and Genomes (KEGG) pathway enrichment analyses were performed. Finally, a potential miRNA-mRNA regulatory network was established, another dataset was used to detect the candidate target gene expression levels, and two relatively meaningful miRNA-mRNA pairs were experimentally verified. In summary, our findings reveal the potential comprehensive mechanisms of miRNA-mRNA regulatory axes in the pathogenesis of bleomycin-induced ALI and a potential ALI-related miRNA-mRNA regulatory network. The flowchart of our study is depicted in Fig. 1.

## Results

### Searching miRNA datasets to identify DE-miRNAs

**Figure 2:**
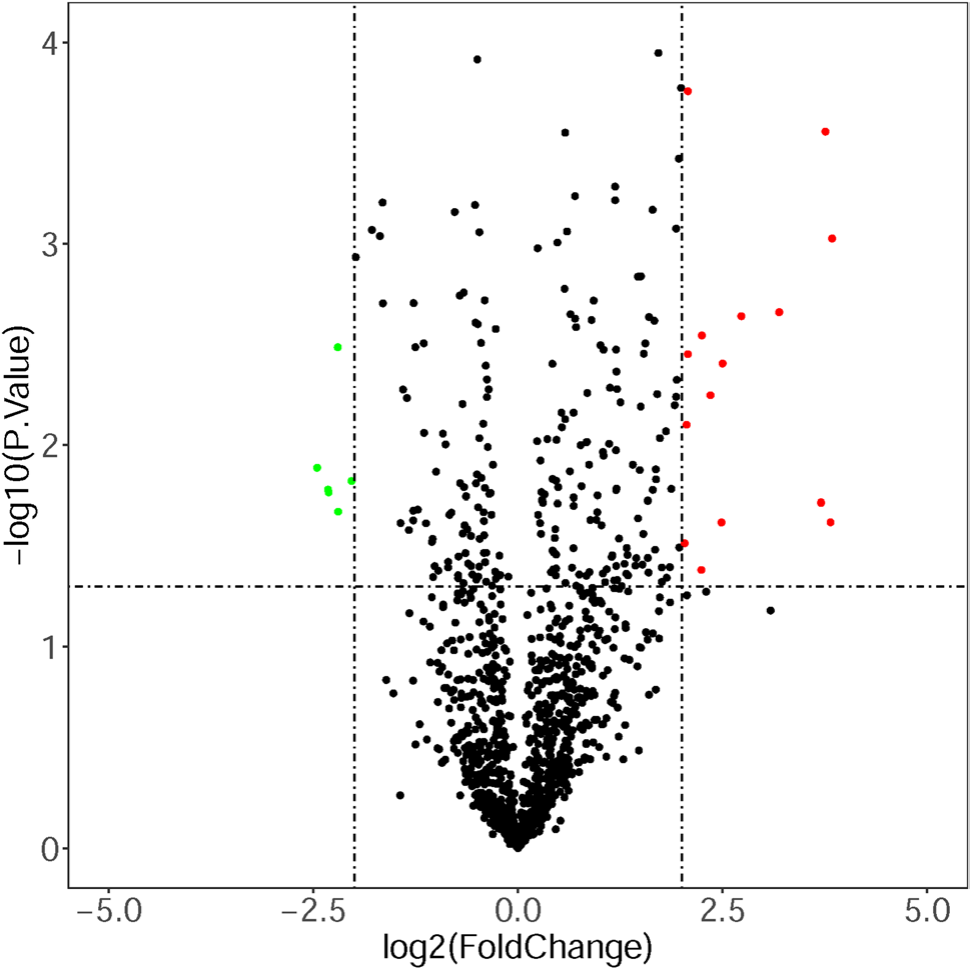
The DE-miRNAs between bleomycin-treated samples and control samples. A |log2FC|> 2 and P < 0.05 were set as the thresholds for identifying DE-miRNAs. The red and green dots represent the upregulated and downregulated miRNAs in bleomycin-treated samples, respectively; the black dots represent miRNAs that were not differentially expressed between the bleomycin-treated samples and control samples.

A dataset (GSE147138) from the Gene Expression Omnibus (GEO) was selected to screen DE-miRNAs between bleomycin-treated samples and control samples. After variance analysis and setting |log2-fold change (FC)|> 2 and P < 0.05 as the thresholds for identifying DE-miRNAs, 15 upregulated DE-miRNAs (mmu-miR-298-5p, mmu-miR-196a-5p, mmu-miR-21a-3p, mmu-miR-96-3p, mmu-miR-7b-5p, mmu-miR-470-5p, mmu-miR-302d-3p, mmu-miR-743b-3p, mmu-miR-871-5p, mmu-miR-871-3p, mmu-miR-881-3p, mmu-miR-465b-5p, mmu-miR-465c-5p, mmu-miR-3092-3p, mmu-miR-344e-3p) and 6 downregulated DE-miRNAs (mmu-miR-448-5p, mmu-miR-451a, mmu-miR-486a-5p, mmu-miR-486a-3p, mmu-miR-486b-5p, mmu-miR-486b-3p) were identified. The volcano plot of the DE-miRNAs is shown in Fig. 2.

### Prediction of potential DE-miRNA target genes

**Figure 3:**
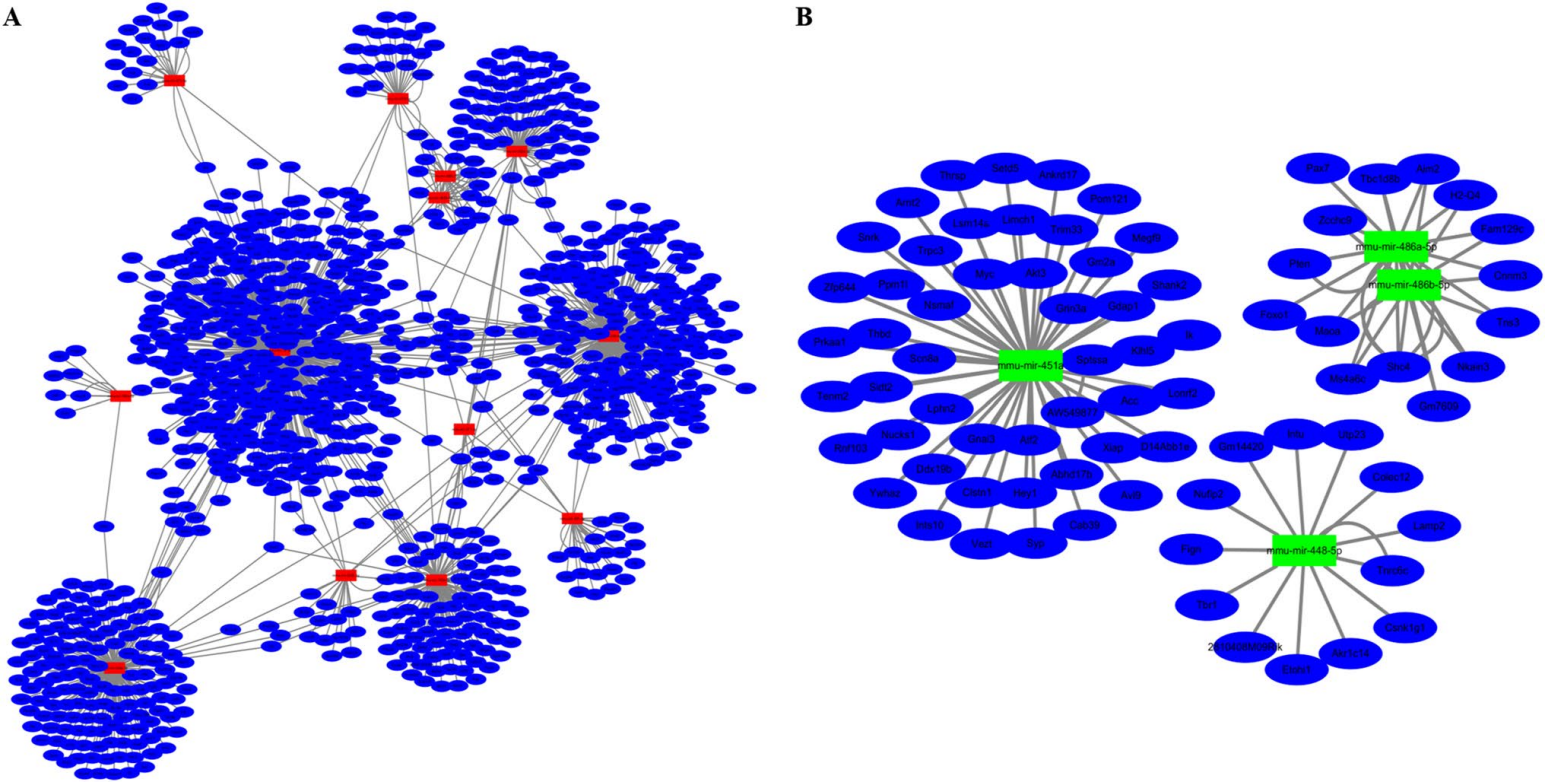
Predicted target genes of the DE-miRNAs. (**A**) Upregulated DE-miRNA-target gene network constructed using miRNet. The red rectangles represent the upregulated DE-miRNAs, and the blue ellipses represent the target genes. (**B**) Downregulated DE-miRNA-target gene network constructed using miRNet. The green rectangles represent the downregulated DE-miRNAs, and the blue ellipses represent the target genes.

We used the miRNet database to predict the potential target genes of the DE-miRNAs, as miRNAs exert their biological effects mainly by directly targeting the 3’ untranslated regions of mRNAs. The potential target genes for the upregulated DE-miRNAs included 1068 genes associated with 13 miRNAs (see Supplementary File 1A), and the potential target genes for the downregulated DE-miRNAs included 76 genes associated with 4 miRNAs (see Supplementary File 1B). The upregulated DE-miRNA-target gene network was established and is presented in Fig. 3A, and the downregulated DE-miRNA-target gene network was established and is presented in Fig. 3B. Additionally, the degrees of target genes for the DE-miRNAs are listed in Table 1.

**Table 1:** Degrees of the target genes of the DE-miRNAs.

	miRNA ID	Degree
Upregulated DE-miRNAs	mmu-mir-7b-5p	468
	mmu-mir-298-5p	300
	mmu-mir-344e-3p	157
	mmu-mir-743b-3p	124
	mmu-mir-302d-3p	77
	mmu-mir-881-3p	28
	mmu-mir-470-5p	28
	mmu-mir-465b-5p	25
	mmu-mir-465c-5p	25
	mmu-mir-871-5p	23
	mmu-mir-3092-3p	20
	mmu-mir-196a-5p	10
	mmu-mir-871-3p	5
Downregulated DE-miRNAs	mmu-mir-451a	50
	mmu-mir-486a-5p	18
	mmu-mir-448-5p	14
	mmu-mir-486b-5p	14

### Searching mRNA datasets to identify DE-mRNAs

**Figure 4:**
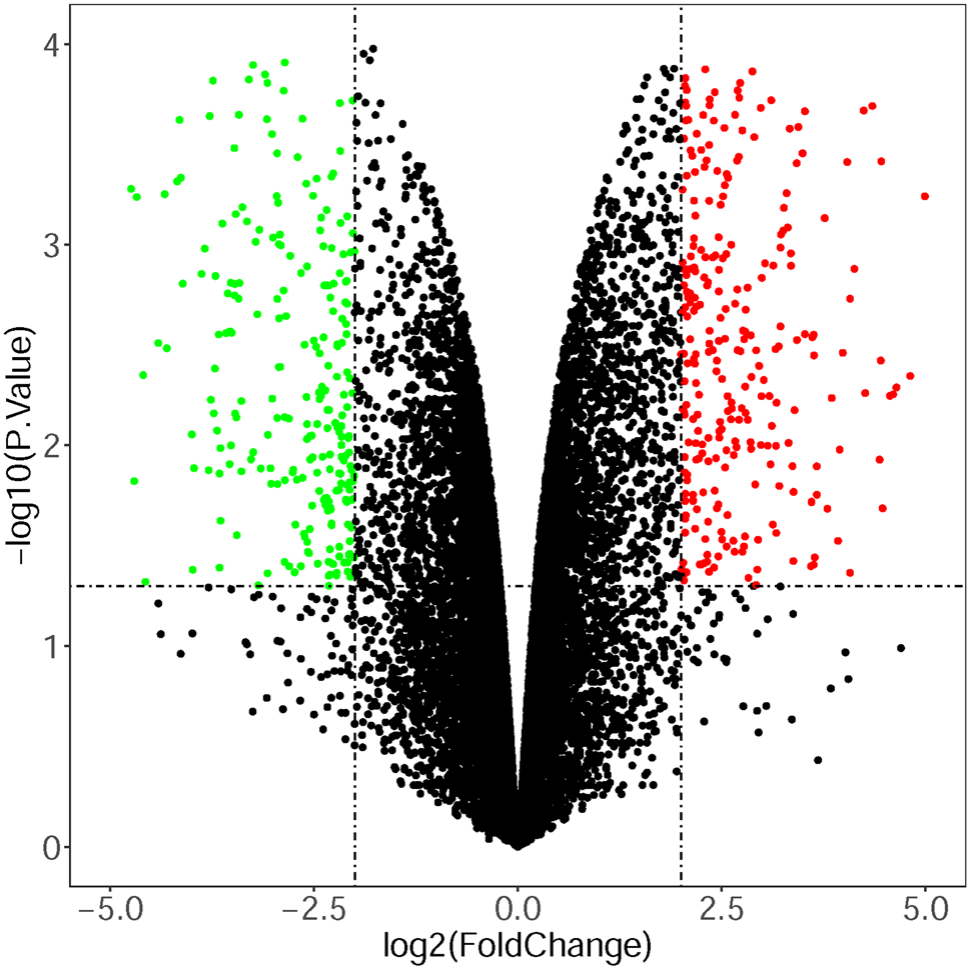
The DE-mRNAs between bleomycin-treated samples and control samples. A |log2FC|> 2 and P < 0.05 were set as the thresholds for identifying DE-mRNAs. The red and green dots represent the upregulated and downregulated mRNAs in the bleomycin-treated samples, respectively; the black dots represent mRNAs that were not differentially expressed between the two groups.

To improve the reliability of our subsequent analysis of the target genes of the screened DE-miRNAs, we searched GEO datasets focusing on mRNA expression. One dataset (GSE123808) was selected to screen DE-mRNAs between bleomycin-treated samples and control samples. Series matrix files were downloaded from the GEO dataset (see Supplementary File 2A). Applying the RGUI and limma packages for analysis of variance, different mRNAs were identified (see Supplementary File 2B). The |log2FC|> 2 and P < 0.05 parameters were set as the thresholds for identifying DE-mRNAs. Finally, 261 downregulated DE-mRNAs (see Supplementary File 2C) and 287 upregulated DE-mRNAs (see Supplementary File 2D) were identified. The volcano plot of the DE-mRNAs is shown in Fig. 4.

### Identification of candidate target genes

**Figure 5:**
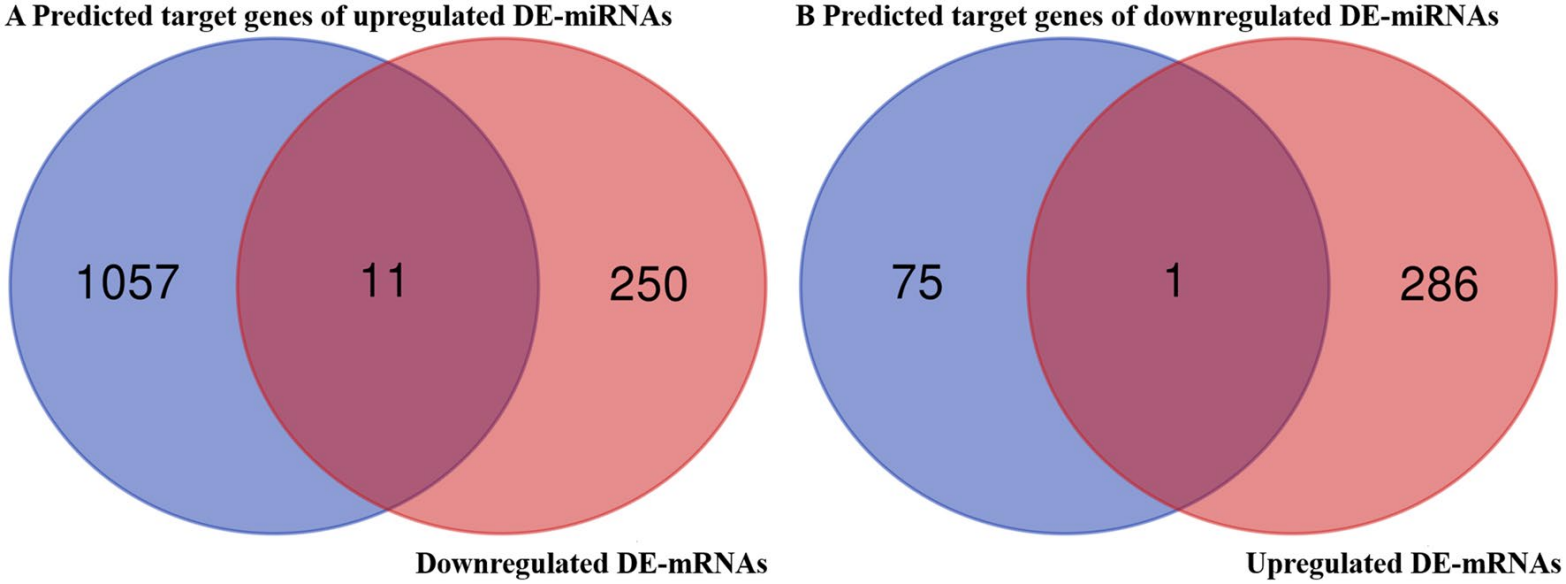
Identification of candidate target genes. (**A**) The intersection of the predicted target genes of the upregulated DE-miRNAs and downregulated DE-mRNAs. (**B**) The intersection of the predicted target genes of the downregulated DE-miRNAs and upregulated DE-mRNAs.

It is widely acknowledged an inverse relationship exists between miRNAs and mRNA target genes. We conducted a combined analysis of 261 downregulated DE-mRNAs and 1068 predicted target genes of the upregulated DE-miRNAs, and 11 candidate target genes of the upregulated DE-miRNAs were further screened out (Fig. 5A, Table 2). We conducted a combined analysis of 287 upregulated DE-mRNAs and 76 predicted target genes of the downregulated DE-miRNAs, and 1 candidate target gene was further screened out (Fig. 5B, Table 3).

**Table 2:** Candidate target genes of the upregulated DE-miRNAs.

Candidate target genes	Upregulated DE-miRNAs
Ugt2b35	mmu-mir-344e-3p
Stk11ip	mmu-mir-7b-5p
Gria1	mmu-mir-7b-5p
Cplx1	mmu-mir-7b-5p
Necab1	mmu-mir-7b-5p
Ces1g	mmu-mir-298-5p
Fzd1	mmu-mir-344e-3p
Senp5	mmu-mir-881-3p
	mmu-mir-298-5p
Ddx3y	mmu-mir-871-5p
Sema4g	mmu-mir-7b-5p
Cadm2	mmu-mir-3092-3p

**Table 3:** Candidate target genes of the downregulated DE-miRNAs.

Candidate target genes	Downregulated DE-miRNAs
Shc4	mmu-mir-486a-5p
	mmu-mir-486b-5p

### Construction of the PPI network

We mapped these candidate target genes into the STRING database, setting the research species as "*Mus musculus*", to construct the PPI network. When the lowest interaction score was set to 0.15, 7 candidate target genes of the DE-miRNAs in the network were predicted to have protein interactions (five disconnected nodes in the network), and 5 edges represented the interactions between proteins (Supplementary Figure S1).

### GO function and KEGG pathway enrichment analyses

GO biological process (BP) functional enrichment analysis showed that the candidate target genes were significantly enriched for membranous septum morphogenesis, synaptic vesicle fusion to the presynaptic active zone membrane, planar cell polarity pathway involved in neural tube closure, stem cell differentiation, regulation of the establishment of planar polarity involved in neural tube closure, vesicle fusion to the plasma membrane, regulation of the postsynaptic cytosolic calcium ion concentration, establishment of vesicle localization, establishment of the planar polarity involved in neural tube closure, and the Wnt signaling pathway involved in midbrain dopaminergic neuron differentiation, among others (See Supplementary File 3A). The top 20 GO BP enrichment terms ranked by their adjusted (adj) P values are shown in Supplementary Figure S2A.

GO molecular function (MF) function enrichment analysis showed that the candidate target genes were significantly enriched for PDZ domain binding, Wnt-activated receptor activity, ionotropic glutamate receptor activity, syntaxin-1 binding, frizzled binding, glutamate receptor activity, Wnt protein binding, transmitter-gated ion channel activity involved in the regulation of postsynaptic membrane potential, neurotransmitter receptor activity involved in the regulation of postsynaptic membrane potential, and postsynaptic neurotransmitter receptor activity, among others (see Supplementary File 3B). The top 20 GO MF enrichment terms ranked by their adj P values are shown in Supplementary Figure S2B.

GO cellular component (CC) function enrichment analysis showed that the candidate target genes were significantly enriched for the dendritic spine membrane, Wnt signalosome, postsynaptic membrane, AMPA glutamate receptor complex, dendrite membrane, calyx of Held, glutamatergic synapse, axon part, SNARE complex, and synaptic membrane, among others (see Supplementary File 3C). The top 20 GO CC enrichment terms ranked by their adj P values are shown in Supplementary Figure S2C.

KEGG pathway enrichment analysis of the candidate target genes was then conducted. The candidate target genes were significantly enriched for the breast cancer, gastric cancer, hepatocellular carcinoma, nicotine addiction, neurodegeneration-multiple diseases, long-term depression, basal cell carcinoma, long-term potentiation, amphetamine addiction, and prolactin signaling pathways (see Supplementary File 3D). The top 20 KEGG pathways enriched ranked by their adj P values are shown in Supplementary Figure S2D.

### Identification of a potential miRNA-mRNA regulatory network

**Figure 6:**
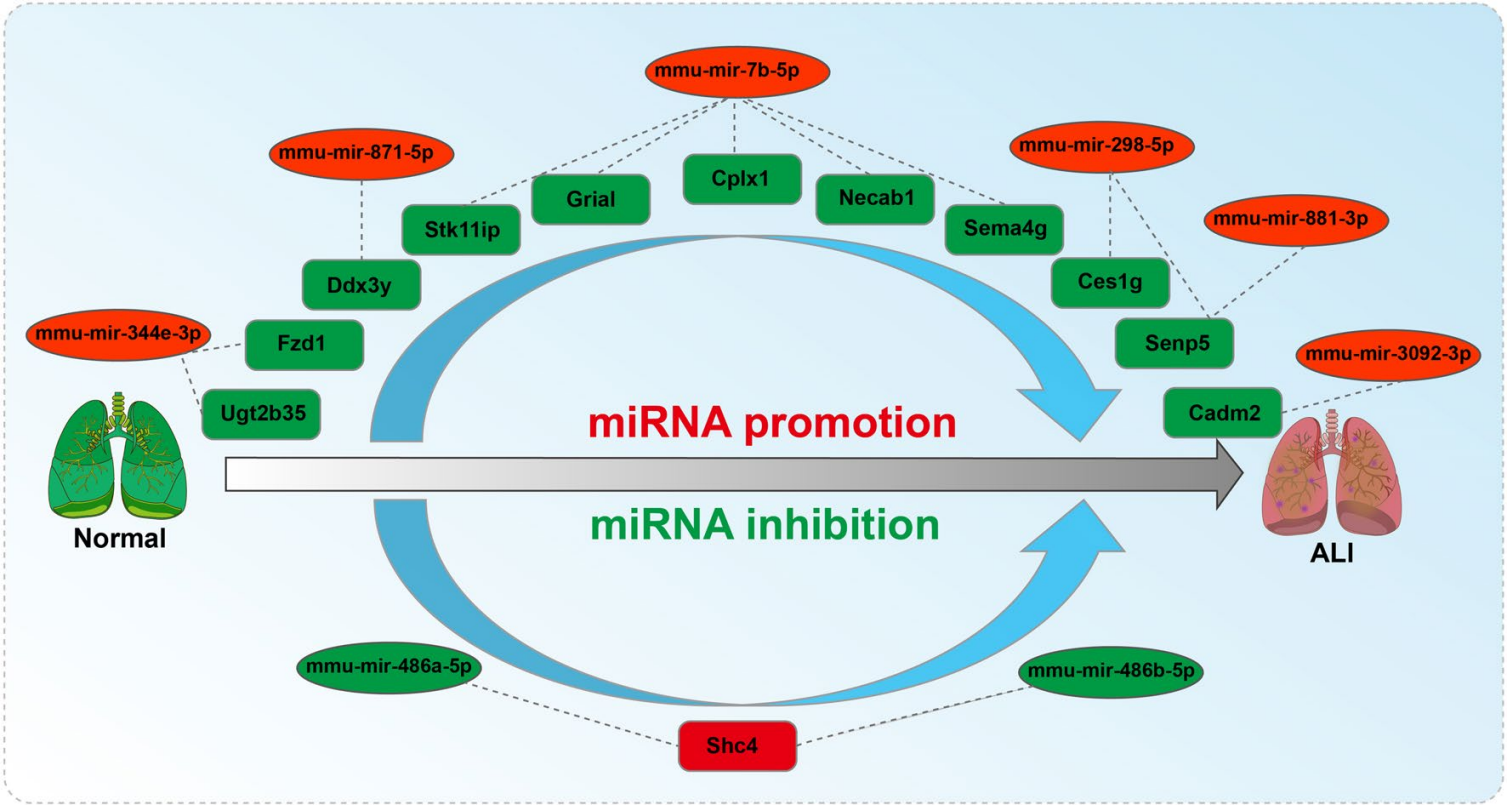
The potential miRNA-mRNA regulatory network of ALI. Upregulated miRNA and downregulated mRNA regulatory network axes included mir-344e-3p-Ugt2b35/Fzd1, mir-7b-5p-Stk11ip/Gria1/Cplx1/Necab1/Sema4g, mir-298-5p-Ces1g/Senp5, mir-881-3p-Senp5, mir-871-5p-Ddx3y and mir-3092-3p-Cadm2; the downregulated miRNA and upregulated mRNA regulatory network axes included mir-486a-5p-Shc4 and mir-486b-5p-Shc4.

According to the miRNA and candidate target gene pairs analyzed above (Tables 2, and 3), we found a link between miRNAs and target genes, and the potential miRNA-mRNA (target gene) regulatory network related to the development of bleomycin-induced ALI in mice was constructed as shown more intuitively in Fig. 6.

### Searching the datasets and validating the candidate target gene expression levels

**Figure 7:**
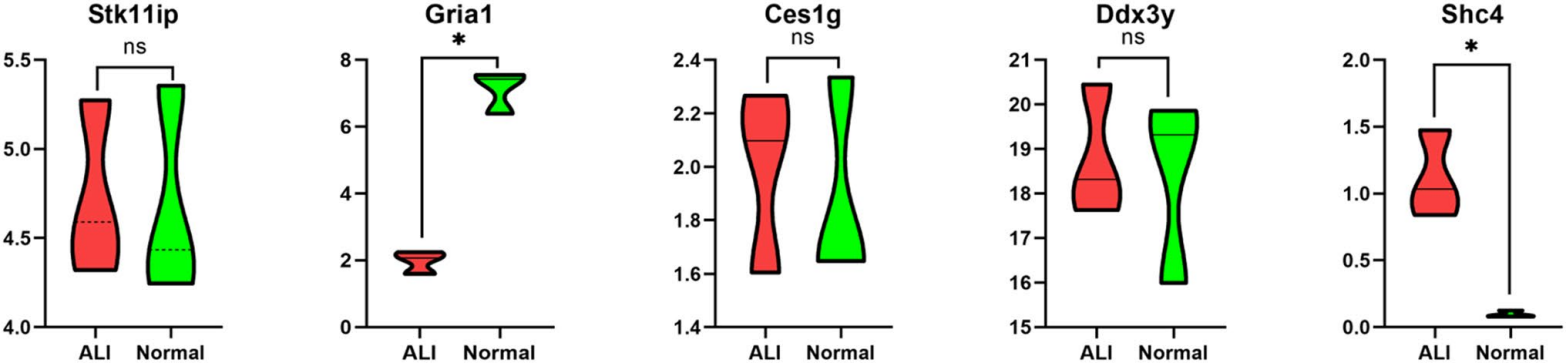
Expression levels of target genes from the GSE109913 dataset. The expression levels of Gria1 were significantly lower in bleomycin-treated ALI samples than in normal control samples, and the expression levels of Shc4 were significantly higher in bleomycin-treated ALI samples than in normal control samples. ns: no significance, *P < 0.05.

To make the validation more credible, we randomly selected a dataset that met the inclusion criteria. Finally, GSE109913 was selected for subsequent analysis. The expression levels of five candidate target genes from the GSE109913 dataset were determined and are shown in Fig. 7. In the GSE109913 dataset, the expression level of Gria1 was significantly lower in bleomycin-treated ALI samples than in normal control samples (P < 0.05), and the expression level of Shc4 was significantly higher in bleomycin-treated ALI samples than in normal control samples (P < 0.05).

Analysis of target gene expression demonstrated the inhibitory effect of Gria1 and the promotional effect of Shc4 on ALI. Based on this preliminary validation, more accurate potential miRNA-mRNA regulatory axes contributing to ALI were established, including the mmu-mir-7b-5p-Gria1, mmu-mir-486a-5p-Shc4 and mmu-mir-486b-5p-Shc4 regulatory pathways, which could first be further studied in clinical and basic experiments.

### Experimental validation of a bleomycin-induced ALI model

**Figure 8:**
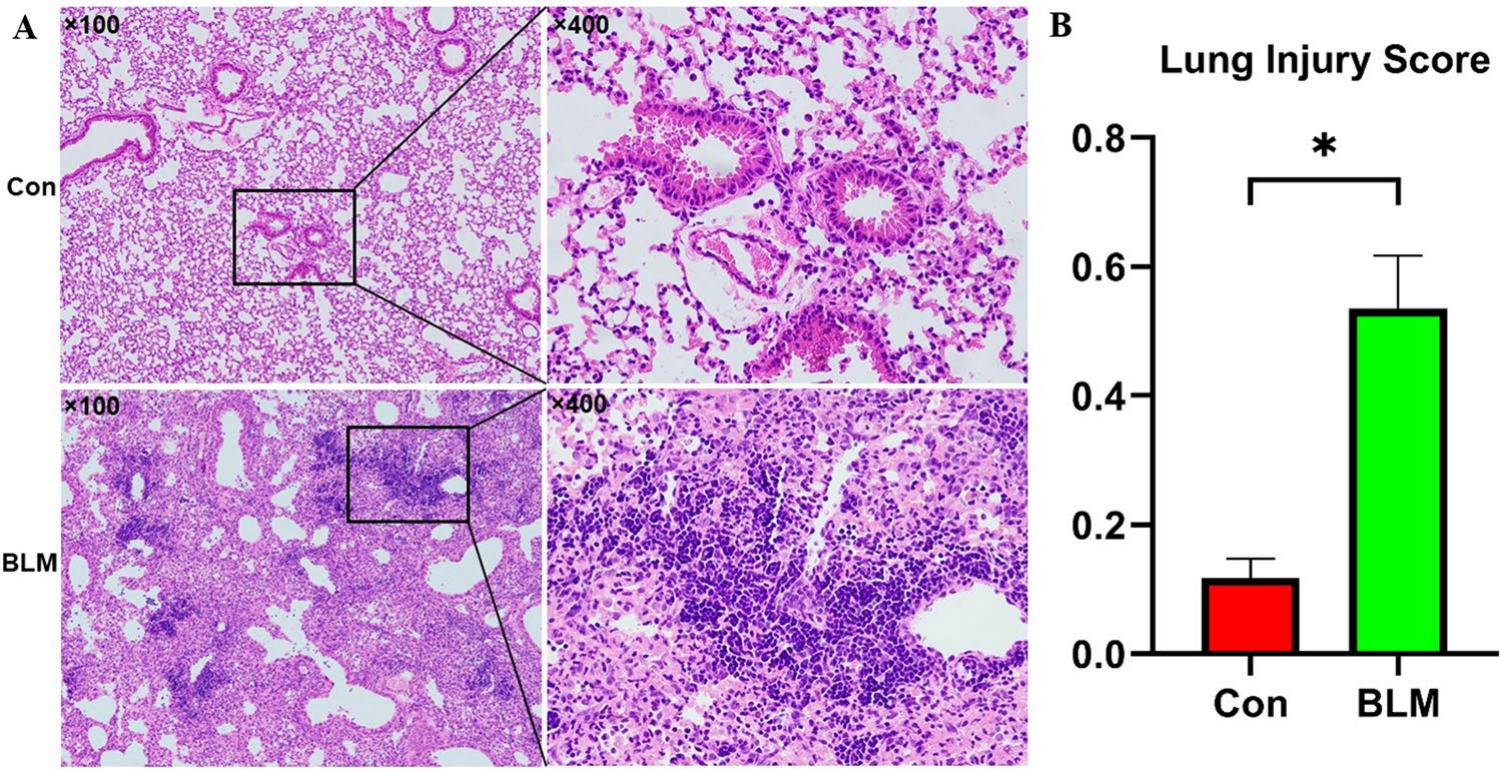
Hematoxylin and eosin (HE) staining and acute lung injury scores of lung samples. (**A**) Representative HE staining images of lung tissues. Scale bar, 100 × magnification in the left panels and 400 × magnification in the right panels. (**B**) The lung injury scores were calculated based on HE staining. n = 6 per group. *P < 0.05.

To further validate the prediction results, we constructed a bleomycin-induced ALI mouse model by intratracheally administering 5 mg/kg bleomycin. Hematoxylin and eosin (HE) staining of lung sections from the bleomycin-treated groups showed comprehensive features of morphological damage, such as congestion, hemorrhaging, thickening of the alveolar walls and infiltration of inflammatory cells, especially neutrophils, while no histological defects were observed in the phosphate-buffered saline (PBS) treated lungs (Fig. 8A). Compared with that of the control group, the lung injury score of the bleomycin-treated group was higher (Fig. 8B).

**Figure 9:**
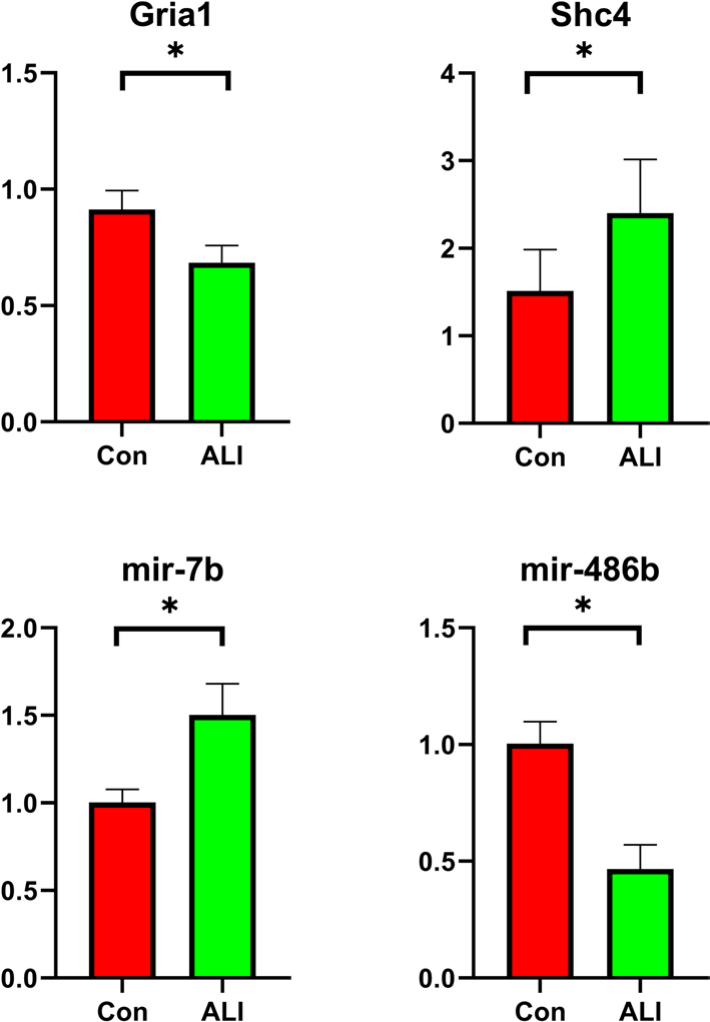
Experimental validation of a bleomycin-induced ALI model by real-time PCR. The expression of miR-7b was significantly upregulated and that of the Gria1 gene was downregulated in the ALI groups. The expression of miR-486b was significantly downregulated and that of the Shc4 gene was upregulated in the ALI groups. n = 6 per group. *P < 0.05.

Then, we explored the expression of miR-7b and its target gene Gria1 and miR-486b and its target gene Shc4 in lung tissues using real-time polymerase chain reaction (PCR) (Fig. 9). Consistent with the predicted results, the experimental validation showed that the expression of miR-7b was significantly upregulated and that of the Gria1 gene was downregulated in the ALI groups (P < 0.05). In addition, the expression of miR-486b was significantly downregulated and that of Shc4 was upregulated in the ALI groups (P < 0.05).

## Discussion

ALI/ARDS is a life-threatening clinical condition associated with multiple symptoms and influenced by numerous factors^1,2^. Functional genomics approaches have provided novel insights into ALI/ARDS, a complex trait that requires both a severe environmental insult and an individual predisposition^15^. To date, the only study showing a link between the miRNA-mRNA regulatory network and ARDS was a study that induced ARDS in rats by using saline lavage and mechanical ventilation^11^.

As an antitumor antibiotic, bleomycin can form complexes with oxygen and iron to break DNA strands, resulting in the secretion of oxygen free radicals and cell apoptosis^16^. During the process of cell damage and apoptosis, a number of cytotoxic factors, such as reactive oxygen species (ROS) and nitrogen (NO) inflammatory factors, are generated in the lungs and can directly damage cells through lipid and protein oxidation^17^. Therefore, bleomycin has been widely used in animal studies to model pulmonary fibrosis and ALI/ARDS^13,14,18^. The bleomycin-induced ALI mouse model is widely applied because it is characterized by an inflammatory response and alveolar epithelia leading to excessive matrix deposition^12–14^. However, no microRNA-mRNA regulatory network of bleomycin-induced ALI in mice has been constructed.

In this study, we searched the GEO database and conducted differential expression analysis using miRNA and mRNA datasets. Finally, six upregulated DE-miRNAs and two downregulated DE-miRNAs were identified. Some of the screened DE-miRNAs were consistent with previous results. MiR-344 was also identified as an upregulated miRNA in a rat model of ARDS that inversely correlated with the expression of their predicted targets, such as Aco2, Mdh1 and Eif2ak1^11^. MiR-7b was previously shown to be upregulated in the ALI/ARDS model^19,20^. Silencing the lncRNA MEG3 augments the binding of miR-7b to NLRP3 and downregulates NLRP3 expression, which ultimately improves endotoxin-induced ALI^21^. Although there are no direct reports of miR-298 in ALI/ARDS, miR-298 was predicted to bind with high affinity to the 5’UTR of the SARS-CoV-2 genome, and SARS-CoV-2 can cause ARDS^22^. MiR-298 was also identified as a potential regulator of the NOD-dependent *Tnf-α* and *Il-6* mRNA levels in pulmonary endothelial cells, which represents the vital pathogenesis of ARDS^23^. In an LPS-induced ALI mouse model, the miR-486-5p level was significantly higher than that in the controls^24^. However, in bleomycin-induced ALI, miR-486-5p was shown to be downregulated^25^. Hence, our findings provide a basis for the use of miRNAs as biomarkers or targets for miRNA-based pharmacological therapies for ALI.

After integrating the DE-mRNAs and target genes of the DE-miRNAs, multiple candidate genes were screened, including 11 candidate target genes of the upregulated DE-miRNAs and 1 candidate target gene of the downregulated DE-miRNAs. Then, a PPI network was constructed to analyze the protein interactions of these target genes. In STRING, each PPI is annotated with one or more 'scores'. These scores are indicators of confidence. All scores rank from 0 to 1, with 1 being the highest possible confidence. There are two types of scores: the “normal” score, and the “transferred” score. The latter is computed from data that is not originally observed in the organism of interest, but instead in some other organism and then transferred via homology/orthology. In this study, we mainly studied miRNA-mRNA interaction. There is indeed little evidence for interactions between potentially related target genes (proteins). Our PPI network results show interactions between possible target genes (proteins) for future in-depth studies. We selected with different thresholds to establish confidence of PPI. When we chose a higher threshold, there were fewer confidence of PPI. For a more comprehensive analysis, we chose a lower threshold score value 0.15.

GO BP functional enrichment analysis showed that the candidate target genes were significantly enriched for membranous septum morphogenesis, synaptic vesicle fusion to the presynaptic active zone membrane, planar cell polarity involved in neural tube closure, stem cell differentiation, and regulation of the establishment of planar polarity involved in neural tube closure. GO MF functional enrichment analysis showed that the candidate target genes were significantly enriched for PDZ domain binding, Wnt-activated receptor activity, ionotropic glutamate receptor activity, syntaxin-1 binding, and frizzled binding. GO CC functional enrichment analysis showed that the candidate target genes were significantly enriched for the dendritic spine membrane, Wnt signalosome, postsynaptic membrane, AMPA glutamate receptor complex, and dendrite membrane. KEGG pathway enrichment analysis showed that candidate target genes were significantly enriched for pathways related to breast cancer, gastric cancer, hepatocellular carcinoma, nicotine addiction and multiple neurodegeneration diseases. Some of these functional enrichment and pathways are closely related to ALI/ARDS, and some of these genes have been identified to act as pivotal modulators. For example, Fzd1 expression was decreased in the lungs of rats with endotoxic shock, and decreased Fzd1 expression may hinder the sensitivity of Wnt3a/β-catenin signaling to regulate inflammatory responses^26^. Shc4 was shown to enhance intracellular antioxidant defense via the nuclear factor erythroid 2-related factor 2 (Nrf2)/heme oxygenase-1 (HO-1) signaling pathway, which was associated with the oxidative stress response in ALI^27^.

The upregulated miRNA and downregulated mRNA regulatory network constructed herein included mir-344e-3p-Ugt2b35/Fzd1, mir-7b-5p-Stk11ip/Gria1/Cplx1/Necab1/Sema4g, mir-298-5p-Ces1g/Senp5, mir-881-3p-Senp5, mir-871-5p-Ddx3y and mir-3092-3p-Cadm2, and the downregulated miRNA and upregulated mRNA regulatory network included mir-486a-5p-Shc4 and mir-486b-5p-Shc4. There are still relatively few reports on these regulatory networks, and ALI-related research has been particularly limited. As a result, these miRNAs and target genes can be combined to perform in-depth studies and thereby identify potential targets for the treatment of related diseases. Thus, further research on this potential ALI-related miRNA-mRNA regulatory network is warranted to verify the relevant mechanism. Almost none of these miRNA-mRNA pairs in the network potentially contributing to the pathogenesis of ALI have been studied, which is of importance for exploring and developing novel mechanisms and therapeutic targets.

To enhance the applicability of our data, we first used datasets including bleomycin-treated samples and control samples to further select suitable pathways to study. In the GSE109913 dataset, the expression levels of Gria1 were significantly lower in bleomycin-induced ALI tissues than in normal tissues, and the expression levels of Shc4 were significantly higher in bleomycin-induced ALI tissues than in normal tissues. Then, we constructed a bleomycin-induced ALI mouse model, which was confirmed by the HE staining of lung sections. Furthermore, we explored the expression levels of miR-7b and its target gene Gria1 and of miR-486b and its target gene Shc4 in lung tissues by real-time PCR. Fortunately, the experimental validation showed that the expression of miR-7b was significantly upregulated while that of the Gria1 gene was downregulated in the ALI groups; the expression of miR-486b was significantly downregulated and that of the Shc4 gene upregulated was in the ALI groups. Although miR-7b was upregulated in the ALI/ARDS model^19,20^, the predicted target genes of miR-7b are IRS2, OXR1, GSK3B, and NFAT5. Here, we identified a new miRNA-mRNA regulatory pathway (miR-7b/Gria1), which was preliminarily verified in a bleomycin-induced ALI mouse model. miR-486b and Shc4 have been shown to be related to oxidative stress, but the miR-486b/Shc4 pathway has not yet been confirmed. Therefore, these gene-related miRNA-mRNA regulatory pathways should be further studied in basic experiments.

Although a potential miRNA-mRNA regulatory network was constructed in this study, there are still some limitations. First, we utilized only one miRNA dataset and one mRNA dataset, and the number and sample sizes of the datasets included in this study were small. Second, we screened out DE-miRNAs and DE-mRNAs from a web database with data from multiple sources to avoid the limitations of a single-center study as much as possible, but a single study to validate and screen the constructed regulatory network is still needed. It is best to verify both miRNAs and mRNAs in the same set of samples. We used only the GSE109913 dataset to preliminarily validate gene expression. On this basis, our next studies will further validate and further explore the underlying mechanisms to find effective interventions to target the established regulatory network. Finally, as we further validated the gene expression and regulatory network, we explored the expression of miR-7b and its target gene Gria1 and miR-486b and its target gene Shc4 in lung tissues using real-time PCR. Further studies on the other miRNAs and target genes are needed in the future.

In conclusion, we herein reveal a potential comprehensive mechanism of miRNA-mRNA regulatory axes in the pathogenesis of bleomycin-induced ALI and establish a potential ALI-related miRNA-mRNA regulatory network, which may provide a basis for basic and clinical research on ALI and advance its treatment.

## Materials and methods

### Searching and screening of datasets

We searched datasets focusing on miRNAs, mRNAs and genes in the GEO dataset (https://www.ncbi.nlm.nih.gov/gds/). Taking miRNA expression as an example, the retrieval strategy was as follows: (("micrornas"[MeSH Terms] OR microRNA [All Fields]) AND ("bleomycin"[MeSH Terms] OR bleomycin [All Fields])) AND "*Mus musculus*"[porgn]. We included datasets based on bleomycin-induced mice and datasets containing bleomycin-treated lung tissue samples and control lung tissue samples. One dataset (GSE147138) met the inclusion criteria mentioned above and was selected for subsequent analysis. The dataset contained bleomycin-treated samples (C57Bl/6 mice received one intratracheal administration of bleomycin in PBS) and control samples (C57Bl/6 mice received one intratracheal administration of PBS alone). Dataset GSE147138 was based on the GPL21103 Illumina HiSeq 4000 platform (*Mus musculus*). Basic information about the dataset is provided in Supplementary File 4.

### Identification of DE-miRNAs

The miRNA expression data (GSE147138) were downloaded from the National Center for Biotechnology Information (NCBI) GEO (see Supplementary File 5). By comparing bleomycin-treated samples and control samples, DE-miRNAs were identified using the RGUI 4.0.3 and limma packages based on |log2FC|> 2 and P value < 0.05 as the thresholds^28^.

### Prediction of potential target genes of DE-miRNAs

An integrated platform linking miRNAs, their targets and their functions named miRNet (https://www.mirnet.ca/) was used to predict the downstream target genes of the screened DE-miRNAs^29–31^. The screened upregulated and downregulated DE-miRNAs were entered into the web platform, and the data of the potential target genes of the upregulated and downregulated DE-miRNAs were downloaded. Then, these data were input into Cytoscape 3.6.0 software to access the DE-miRNA-target gene network^32^. Using the "Network Analyzer" tools of the software, the data were subjected to topology analysis, and the degrees of target genes for the DE-miRNAs were finally identified.

### Identification of DE-mRNAs and candidate target genes

GSE123808 was based on the GPL23479 BGISEQ-500 platform (*Mus musculus*), and basic information about this dataset is provided in Supplementary File 4. Setting |log2FC|> 2 and P value < 0.05 as the thresholds, DE-mRNAs were identified using the RGUI and limma packages^28^. Then, we analyzed the DE-mRNAs and predicted target genes of DE-miRNAs in combination, and candidate target genes were further screened.

### Construction of the PPI network

The candidate target genes were introduced into the STRING database (https://string-db.org/). STRING is an ELIXIR Core Data web server that retrieves and displays repeatedly occurring gene neighborhoods^33–35^. After adding the candidate target genes into the database, a PPI network was constructed. The research species was defined as "*Mus musculus*", the lowest interaction score was set to 0.15, and the remaining parameters were set to the default settings. Nodes represented target genes, and edges represented the interactions between the target genes in the PPI network.

### GO function and KEGG pathway enrichment analyses

The RGUI 4.0.3 and org.Hs.eg.db packages were applied to obtain the entrezIDs of the candidate target genes. RGUI and the clusterProfiler package were used to perform GO function enrichment analysis, which included BP, MF and CC, as well as KEGG pathway enrichment analysis^36,37^.

### Identification of a potential miRNA-mRNA regulatory network and validation of target gene expression levels

According to the miRNA and candidate target gene pairs analyzed, we established a link between miRNAs and candidate target genes to identify a potential miRNA-mRNA regulatory network. Subsequently, the GEO dataset was used to detect the candidate target gene expression levels. We searched GEO datasets focusing on gene expression and included those based on bleomycin-treated samples and control samples. To make the validation more credible, we randomly selected a dataset that met the inclusion criteria, and GSE109913, which was based on the GPL21103 Illumina HiSeq 4000 platform (*Mus musculus*), was selected for subsequent analysis. Basic information about GSE109913 is provided in Supplementary File 4. We downloaded gene expression data from the GEO dataset and accessed candidate target gene expression data to perform statistical analysis (Supplementary File 6). The expression levels of target genes in the regulatory network were further validated by analyzing the gene expression data downloaded from the GEO dataset. P < 0.05 was considered statistically significant.

### Animal experiments

Six- to eight-week-old male C57BL/6 wild-type mice (Shanghai Laboratory Animal Center, Chinese Academy of Sciences, Shanghai, China) were maintained in a controlled environment and provided water and standard rodent food. The mice were anesthetized with sodium pentobarbital (60 mg/kg) and then administered bleomycin (BLM, Sigma–Aldrich Co. LLC., USA) dissolved in PBS via a single intratracheal instillation at a dose of 5 mg/kg body weight in a volume of 50 μl to induce ALI; mice in the control group received an equal volume of PBS. The mice were anesthetized and sacrificed on day 7 after the bleomycin or PBS injection. The animal experiments were approved by the Animal Ethics Committee of Nantong University on the Use and Care of Animals and were performed in accordance with the committee’s guidelines (ethical approval number, S20210304-019). This study also adhered to the ARRIVE guidelines (https://arriveguidelines.org).

### Histopathological examination

Histological analysis of the left lung was performed. Briefly, immediately after euthanasia, the left lung tissues were collected and fixed in 10% formalin for 24 h, embedded in paraffin, sliced into 5-μm thick sections, and stained with hematoxylin and eosin (HE) for the detection of pathological changes in the lung tissues. Lung injury scores were utilized to evaluate BLM-induced lung injury based on HE images and Matute-Bello’s published criteria in a blinded manner^38^.

### RNA extraction, reverse transcription and real-time quantitative PCR

Total RNA was extracted from the lung tissues of mice using RNAiso Plus (Takara) and reverse-transcribed into complementary DNA using PrimeScript™ RT Master Mix (Takara). mRNA expression levels were quantified using TB Green® Premix Ex Taq™ II (Takara), with GAPDH expression serving as an internal control. Primers with the following sequences were used for real-time PCR: Shc4: forward: 5′-AGC CCA TAC TGG TGC CAT TGA-3′; reverse: 5′-GTT GAA CCA TTG TCC GGT GTG TAG-3′; Gria1: forward: 5′-AGC GGA CAA CCA CCA TCT CTG-3′; reverse: 5′-AAG GGT CGA TTC TGG GAT GTT TC -3′; and GAPDH: forward: 5′-TGC ACC ACC AAC TGC TTA G-3′; reverse: 5′-GGA TGC AGG GAT GAT GTT C-3′. The mir-7b and mir-486b primers and U6 snRNA (internal control) were purchased from RiboBio (Guangzhou, China). miRNA real-time PCR was performed by using the Bulge-loop™ miRNA qRT–PCR Starter Kit (RiboBio, Guangzhou, China) according to the manufacturer's protocol. Data were quantified using the comparative 2 − ΔΔCt method.

### Statistical analysis

Some statistical analyses were automatically performed by the bioinformatic tools on the web platforms mentioned above. We used a series of matrix files downloaded from the GEO dataset analyzed with the RGUI 4.0.3 and the limma packages to identify DE-miRNAs and DE-mRNAs. Only miRNAs and mRNAs with a |log2FC|> 2 and P < 0.05 were considered statistically significant. We used the RGUI 4.0.3 and the org.Hs.eg.db packages to obtain the entrezIDs of the candidate target genes. We used the RGUI and clusterProfiler packages to perform GO functional enrichment analysis, and adjusted P < 0.05 was considered statistically significant. The data of target gene expression levels in GSE109913 dataset, the lung injury scores and miRNA-mRNA pairs expression levels in mouse models were analyzed using IBM SPSS Statistics 25 software and GraphPad Prism 8.0.2 software. Student’s t test or Welch's t test was used to compare two groups. If the data were not normally distributed, the Mann–Whitney U test was used. P < 0.05 was considered significant.

## Supplementary Information

Supplementary Information 1.

Supplementary Information 2.

Supplementary Information 3.

Supplementary Information 4.

Supplementary Information 5.

Supplementary Information 6.

Supplementary Information 7.

Supplementary Information 8.

Supplementary Information 9.

Supplementary Information 10.

Supplementary Information 11.

Supplementary Information 12.

Supplementary Information 13.

Supplementary Information 14.

Supplementary Information 15.

Supplementary Information 16.

## Data Availability

The datasets generated and/or analyzed during the current study are available from the corresponding author upon reasonable request. All data generated or analyzed during this study are included in this published article (and its Supplementary Information files).
