# Supplementary figures and images for: Construction of a potential microRNA and messenger RNA regulatory network of acute lung injury in mice
Source: Sci Rep. 2022 Jan 17;12:777. doi: 10.1038/s41598-022-04800-3 (PMC8763866; doi:10.1038/s41598-022-04800-3)

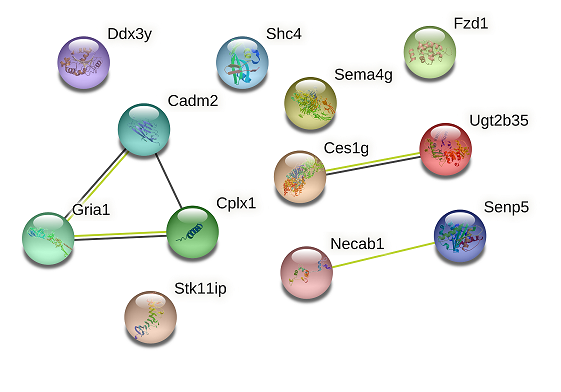

Supplement: Supplementary file 1 — Supplementary Information 1. [file 41598_2022_4800_MOESM1_ESM.tif]

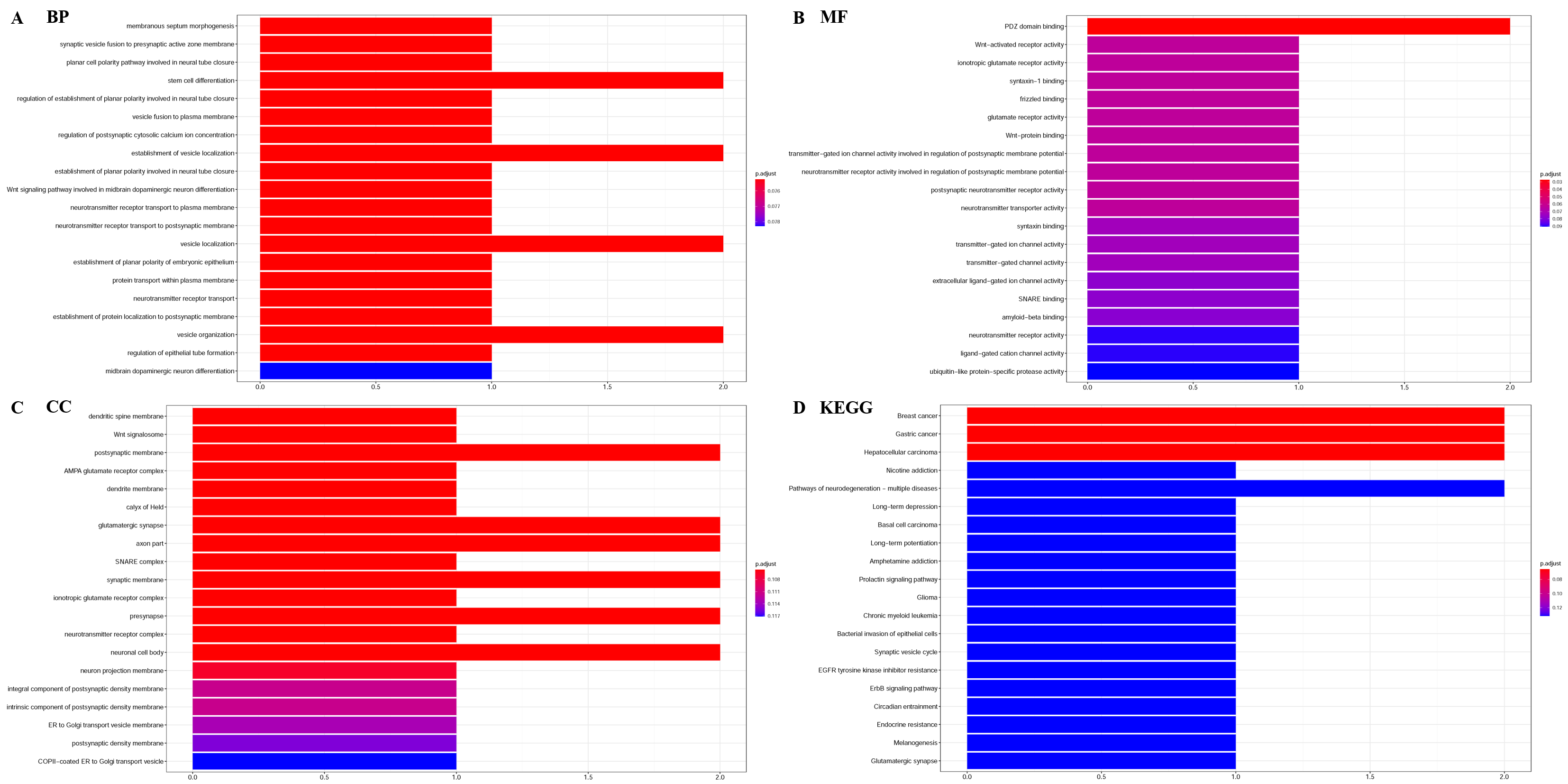

Supplement: Supplementary file 2 — Supplementary Information 2. [file 41598_2022_4800_MOESM2_ESM.tif]
