# Supplementary material for: Construction of a potential microRNA and messenger RNA regulatory network of acute lung injury in mice
Source: Sci Rep. 2022 Jan 17;12:777. doi: 10.1038/s41598-022-04800-3 (PMC8763866; doi:10.1038/s41598-022-04800-3)
Supplement: Supplementary file 16 — Supplementary Information 16. [file 41598_2022_4800_MOESM16_ESM.docx]

**Supplementary information captions**

**Supplementary File 1** Predicted target genes for DE-miRNAs

(A) Predicted target genes for the upregulated DE-miRNAs (1068); (B) Predicted target genes for the downregulated DE-miRNAs (76).

**Supplementary File 2** mRNAs expression data (GSE123808)

(A) mRNA expression data of GSE123808; (B) Different mRNAs of GSE123808; (C) Downregulated DE-mRNAs (261); (D) Upregulated DE-mRNAs (287).

**Supplementary File 3** GO function enrichment and KEGG pathway enrichment analysis

(A) BP function enrichment analysis; (B) MF function enrichment analysis; (C) CC function enrichment analysis; (D) KEGG pathway enrichment analysis.

**Supplementary File 4** Basic information about datasets

**Supplementary File 5** miRNA expression data of GSE147138

**Supplementary File 6** Gene expression data (GSE109913)

**Supplementary Figure S1** PPI network of candidate target genes

Nodes represent target genes and edges represent the interaction between target genes in the PPI network.

**Supplementary Figure S2** Top 20 GO function and KEGG pathway enrichments

(A) The top 20 enriched BP of candidate target genes; (B) The top 20 enriched MF of candidate target genes; (C) The top 20 enriched CC of candidate target genes; (D) The top 20 KEGG pathway enrichments.
